# Structure‐specific effects of lipidated oxytocin analogs on intracellular calcium levels, parental behavior, and oxytocin concentrations in the plasma and cerebrospinal fluid in mice

**DOI:** 10.1002/prp2.290

**Published:** 2017-01-17

**Authors:** Stanislav M. Cherepanov, Shigeru Yokoyama, Akira Mizuno, Wataru Ichinose, Olga Lopatina, Anna A. Shabalova, Alla B. Salmina, Yasuhiko Yamamoto, Hiroshi Okamoto, Satoshi Shuto, Haruhiro Higashida

**Affiliations:** ^1^ Department of Basic Research on Social RecognitionResearch Center for Child Mental DevelopmentKanazawa UniversityKanazawa920‐8640Japan; ^2^ Faculty of Pharmaceutical SciencesHokkaido UniversityKita‐12, Nishi‐6, Kita‐kuSapporo060‐0812Japan; ^3^ Research Institute of Molecular Medicine & PathobiochemistryKrasnoyarsk State Medical University named after Prof. V.F. Voino‐YasenetskyKrasnoyarsk660022Russia; ^4^ Department of Biochemistry and Molecular Vascular BiologyGraduate School of Medical SciencesKanazawa UniversityKanazawa920‐8640Japan; ^5^ Department of Advanced Biological Sciences for Regeneration (Kotobiken Medical Laboratories)Tohoku University Graduate School of MedicineSendai980‐8575Japan; ^6^ Center for Research and Education on Drug DiscoveryHokkaido UniversityKita‐12, Nishi‐6, Kita‐kuSapporo060‐0812Japan

**Keywords:** Autism, CD38, lipidation, oxytocin, oxytocin analog, parental behavior

## Abstract

Oxytocin (OT) is a neuroendocrine nonapeptide that plays an important role in social memory and behavior. Nasal administration of OT has been shown to improve trust in healthy humans and social interaction in autistic subjects in some clinical trials. As a central nervous system (CNS) drug, however, OT has two unfavorable characteristics: OT is short‐acting and shows poor permeability across the blood–brain barrier, because it exists in charged form in the plasma and has short half‐life. To overcome these drawbacks, an analog with long‐lasting effects is required. We previously synthesized the analog, lipo‐oxytocin‐1 (LOT‐1), in which two palmitoyl groups are conjugated to the cysteine and tyrosine residues. In this study, we synthesized and evaluated the analogs lipo‐oxytocin‐2 (LOT‐2) and lipo‐oxytocin‐3 (LOT‐3), which feature the conjugation of one palmitoyl group at the cysteine and tyrosine residues, respectively. In human embryonic kidney‐293 cells overexpressing human OT receptors, these three LOTs demonstrated comparably weak effects on the elevation of intracellular free calcium concentrations after OT receptor activation, compared to the effects of OT. The three LOTs and OT exhibited different time‐dependent effects on recovery from impaired pup retrieval behavior in sires of CD38‐knockout mice. Sires treated with LOT‐1 showed the strongest effect, whereas others had no or little effects at 24 h after injection. These results indicated that LOTs have structure‐specific agonistic effects, and suggest that lipidation of OT might have therapeutic benefits for social impairment.

**Abbreviations**
ASD: autism spectrum disorder
BBB: blood–brain barrier
CSF: cerebro‐spinal fluid
DFM: dimethylformamide
FBS: fetal bovine serum
HBS: HEPES‐buffered saline
HEK: human embryonic kidney
LOT: lipo‐oxytocin
OT: oxytocin
PBS: phosphate‐buffered saline
PVN: paraventricular nucleus
SON: supraoptic nucleus

## Introduction

Oxytocin (OT) and arginine vasopressin (AVP) are nonapeptides that differ in two amino acid residues. These peptides are mainly synthesized in distinct neurons in the paraventricular nucleus (PVN) and supraoptic nucleus (SON) in the hypothalamus (Jin et al. 2007; Higashida 2016). OT and AVP are secreted into the blood and have physiological roles in peripheral organs, but are also present in male and female brains (Neumann 2008; Higashida et al. 2012). Both peptides play critical roles in social recognition and social behavior in mammals, including humans (Kosfeld et al. 2005; Ebstein et al. 2009; Ross et al. 2009; Onaka et al. 2012; Lukas and Neumann 2013; Zoicas et al. 2014; Arakawa et al. 2015; Nagasawa et al. 2015; Zhang et al. 2015). More importantly, OT secretion into the brain is critical to neuronal function of OT in social recognition and behavior (Higashida 2016).

Many methodologies have been developed to address the undesirable pharmacokinetic properties of natural peptide hormones, such as rapid degradation (Egleton and Davis 2005; Popov et al. 2013; Chapman et al. 2013; Prokai‐Tatrai and Prokai 2011; Sciabola et al. 2016). One successful method for elongation of the half‐life in blood, is lipidation, which involves conjugation of a given peptide with long fatty acids (Varamini and Toth 2013; Mohammed et al. 2013; Mäde et al. 2014; Goodwin et al. 2015). Although the effects of lipidation of a parent molecule on blood–brain barrier (BBB) penetration have not been documented, hydrophobic small molecules with a higher log‐*P* values show better penetration across the BBB compared to their parental hydrophilic congeners (Egleton and Davis 2005). On the basis of this hypothesis, we previously synthesized an OT analog, lipo‐oxytocin‐1 (LOT‐1), by conjugating two palmitoyl groups at the amino group of the Cys^1^ residue and the phenolic hydroxyl group of the Tyr^2^ residue (Mizuno et al. 2015). We have determined the effectiveness of LOT‐1 as a central nervous system (CNS) drug in CD157 knockout mice (Lopatina et al. 2014). Intraperitoneal administration of LOT‐1 at an equivalent concentration to OT (nanomolar range) rescued anxiety‐like behavior and social avoidance for a target mouse, even at 24 h after injection (Mizuno et al. 2015). Recently, similar substitutions of OT, in which Leu^8^ is changed to Lys appended with a polyethylene glycol spacer and a palmitoyl group, have been reported (Modi et al. 2016).

Autism spectrum disorder (ASD) is a neurodevelopmental disorder characterized by impairments in social communication and the presence of repetitive behaviors (Lai et al. 2014). In clinical settings, there are few effective treatments for the symptoms of ASD. A great deal of research has focused on the promising candidate neuroendocrine hormone OT for the treatment of social deficits (Yamasue et al. 2012; Hofmann et al. 2015). The data from OT trials in healthy human subjects indicate that OT plays a role in social behaviors (D’Cunha et al. 2011; Lukas et al. 2011; Lukas and Neumann 2013; Elmadih et al. 2014; Zoicas et al. 2014; Zhang et al. 2015; Arakawa et al. 2015). In ASD patients (Macdonald and Macdonald 2010; Munesue et al. 2010; Modi and Young 2012; Lukas and Neumann 2013) OT resulted in recovery of social behavior (Bakermans‐Kranenburg and van I Jzendoorn 2013), although no significant effects were shown in other randomized controlled trial (Guastella et al. 2015). Therefore, it is worth developing OT‐related agents as potential medications for ASD and/or other psychiatric diseases involving social impairment.

We developed CD38 knockout (CD38^−/−^) mice in 2007 to evaluate effects on social behavior (Jin et al. 2007; Higashida 2016; Liu et al. 2016). CD38 is crucial to the release of OT from oxytocinergic neurons in the hypothalamus (Jin et al. 2007; Higashida 2016; Zhong et al. 2016). The CD38^−/−^ mice with reduced OT secretion capacity showed social amnesia (Higashida et al. 2012), as observed in OT or OT receptor‐knockout mice (Caldwell et al. 2016). Interestingly, administration of OT rescued the social impairments in CD38^–/−^ mice (Akther et al. 2013; Higashida and Munesue 2013). In addition, two single‐nucleotide polymorphisms of the CD38 gene were shown to be associated with ASD in human studies performed in the USA, Israel, and Japan (Munesue et al. 2010; Feldman et al. 2011; Sauer et al. 2012; Feldman et al. 2013). We used CD38^−/−^ mice for the evaluation of OT analogs as potential therapeutic drugs for psychiatric diseases with social deficits.

In this study, in addition to LOT‐1, we synthesized lipo‐oxytocin‐2 (LOT‐2) and lipo‐oxytocin‐3 (LOT‐3), which feature the conjugation of only one palmitoyl group at the amino group of the Cys^1^ residue or the phenolic hydroxyl group of the Tyr^2^ residue, respectively. We evaluated and compared the characteristics of these three lipid‐modified molecules. First, the functional effects of OT the analogs on OT receptor stimulation were examined by monitoring intracellular free Ca^2+^ concentrations ([Ca^2+^]_i_) in human embryonic kidney (HEK) cells expressing human OT receptors (Ma et al. 2013). Second, we examined the recovery effect of LOTs on pup retrieval behavior by sires of CD38^−/−^ mice (Liu et al. 2013; Akther et al. 2013). Finally, we measured OT concentrations in the plasma and cerebrospinal fluid (CSF), to examine the long‐lasting effects of LOTs caused by cleavage of the lipids.

## Materials and Methods

### Synthesis of LOT‐1

Synthesis of LOT‐1 was described previously (Mizuno et al. 2015). Briefly, palmitic anhydride (25 mg, 51 *μ*mol) in CH_2_Cl_2_ (0.80 mL) was added to a solution of oxytocin acetate (42 mg, 39 *μ*mol) and triethylamine (17 *μ*L, 0.12 mmol) in dimethylformamide (DMF) (0.80 mL). After removal of the solvent, the residue was washed with Et_2_O, filtered, and dried to give N‐palmitoyloxytocin as a white powder (46 mg). Palmitoyl chloride (17 *μ*L, 56 mmol) in CH_2_Cl_2_ (1.6 mL) was added to a solution of the obtained N‐palmitoyloxytocin white powder (46 mg), triethylamine (15 *μ*L, 110 *μ*mol), and N,N‐dimethylaminopyridine (3.6 mg, 30 *μ*mol) in DMF (1.6 mL). After quenching the reaction was by addition of MeOH, removing the solvent, and washing with Et_2_O, the residue was purified by silica gel column chromatography (silica gel supporting COOH, 50%–100% EtOH in AcOEt) to give LOT‐1 (44 mg, 30 *μ*mol, 79%) as a white powder. The ^1^H‐NMR (400 MHz, DMSO‐d6) values were reported previously (Mizuno et al. 2015). HRMS (ESI) calculated for C_75_H_126_N_12_O_14_ S_2_Na: 1505.8850 [(M + Na)+], found: 1505.8807. The molecular weight was 1483. Purity of LOT‐1 was determined as 98.2% by HPLC (Kinetex 1.7 *μ*C8, 2.1 × 50 mm; A 0.05% aq. Formic acid, B 0.05% formic acid in MeCN; 1–10 min/1%–95% B gradient, 10–20 min/95% B; retention time 9.9 min), where OT (retention time 2.6 min) was below the limit of detection.

### Synthesis of LOT‐2

To a solution of oxytocin acetate (42 mg, 39 *μ*mol) and triethylamine (17 *μ*L, 0.12 mmol) in DMF (0.80 mL) was added palmitic anhydride (25 mg, 51 *μ*mol) in CH_2_Cl_2_ (0.80 mL), and the reaction mixture was stirred for 3 h. After the solvent was removed under reduced pressure, the residue was washed with Et_2_O, filtered, and dried to give the title compound (46 mg, 37 *μ*mol, 95%) as a white powder. ^1^H‐NMR (400 MHz, DMSO‐d6) *δ* 9.15 (1 H, s), 8.66 (1 H, br), 8.27–8.06 (4 H, m), 7.98–7.90 (2 H, m), 7.62 (1 H, br), 7.36 (1 H, s), 7.30 (1 H, s), 7.12–7.05 (4 H, m), 6.90 (1 H, s), 6.81 (1 H, s), 6.62 (2 H, d), 4.96 (1 H, br), 4.69 (1 H, m), 4.56 (1 H, m), 4.39 (1 H, m), 4.32 (1 H, m), 4.17 (1 H, m), 3.96 (1 H, br), 3.88 (1 H, br), 3.68–3.50 (4 H, m), 3.28 (1 H, m), 3.19–3.10 (2 H, m), 3.03–2.93 (2 H, m), 2.78–2.52 (4 H, m), 2.18–1.71 (10 H, m), 1.71–1.35 (6 H, m), 1.31–1.12 (25 H, m), 0.94–0.78 (15 H, m). HRMS (ESI) calculated for C_59_H_96_N_12_O_13_ S_2_Na: 1267.6553 [(M+Na)+], found: 1267.6536. Purity of LOT‐2 was determined as 97.8% by HPLC (Kinetex 1.7 *μ*m C8, 2.1×50 mm; A 0.05% aq. formic acid, B 0.05% formic acid in MeCN; 1–10 min/5–95% B gradient, 10–12 min/95% B; room temperature; detector, 220 nm; injection, 5 *μ*L (1 mg/mL in EtOH); retention time, 7.3 min), where OT (retention time 2.6 min) was below the limit of detection.

### Synthesis of LOT‐3

To a solution of oxytocin acetate (20 mg, 19 *μ*mol) and triethylamine (9.0 *μ*L, 65 *μ*mol) in DMF (2.0 mL) was added Boc_2_O (6.0 *μ*L, 26 *μ*mol) in DMF (0.10 mL), and the reaction mixture was stirred for 4 h. After the solvent was removed under reduced pressure, the residue was washed with Et_2_O, filtered, and dried to give crude Boc‐oxytocin, which was used for the next reaction without further purification. To the crude Boc‐oxytocin (16 mg, 14 *μ*mol), triethylamine (6.0 *μ*L, 43 *μ*mol), and dimethylaminopyridine (1.8 mg, 15 *μ*mol) in DMF (0.70 mL) was added palmitoyl chloride (8.8 *μ*L, 29 *μ*mol) in CH_2_Cl2 (0.70 mL), and the reaction mixture was stirred overnight. After the reaction was quenched with addition of MeOH, the solvent was removed under reduced pressure. The residue was washed with Et_2_O, and purified by silica gel column chromatography (silica gel supporting COOH, 50–100% EtOH in AcOEt) to give the Boc‐Tyr(O‐palmitoyl)‐oxytocin (18 mg, 13 *μ*mol, 93%) as a white powder. The obtained Boc‐Tyr(palmitoyl)‐oxytocin (18 mg, 13 *μ*mol) was dissolved in a mixture solvent of TFA/CH_2_Cl_2_/anisole (0.80 mL/0.10 mL/0.10 mL), and the solution was stirred for 2 h. After the solvent was removed under reduced pressure, the residue was washed with Et_2_O, filtered, and dried to give the title compound (15 mg, 11 *μ*mol, 85%) as a white powder. ^1^H‐NMR (400 MHz, DMSO‐d6) *δ* 8.61–8.48 (2 H, m), 8.37 (1 H, br), 8.29 (1 H, br), 8.06 (1 H, d), 7.91 (1 H, m), 7.74 (1 H, br), 7.44–7.29 (4 H, m), 7.12 (2 H, br), 7.04 (2 H, d), 6.97 (1 H, s), 6.85 (1 H, s), 4.79–4.67 (2 H, m), 4.47 (1 H, m), 4.31 (1 H, m), 4.17 (1 H, m), 4.00–3.91 (2 H, m), 3.85 (1 H, m), 3.69–3.40 (4 H, m), 3.29 (1 H, m), 3.18 (1 H, m), 3.10–2.97 (2 H, m), 2.87 (1 H, m), 2.68–2.50 (4 H, m), 2.18–1.72 (10 H, m), 1.69–1.41 (6 H, m), 1.39–1.09 (25 H, m), 0.98–0.76 (15 H, m). HRMS (ESI) calculated for C_59_H_97_N_12_O_13_ S_2_: 1245.6734 [(M+H)+], found: 1245.6714. The purity of LOT‐3 was determined as 98.8% by HPLC (Kinetex 1.7 *μ* C8, 2.1 × 50 mm; A 0.05% aq. formic acid, B 0.05% formic acid in MeCN; 1–10 min/20–95% B gradient, 10–12 min/95% B; room temperature; detector, 220 nm; injection, 5 *μ*L (1 mg/mL in EtOH); retention time, 5.4 min), where OT (retention time 2.6 min) was below the limit of detection.

### Animals

As described previously (Akther et al. 2013), wild‐type male and female Slc:ICR mice (Institute of Cancer Research of the Charles River Laboratories, Inc., Wilmington, MA) were obtained from Japan SLC, Inc. (Hamamatsu, Japan) through a local distributor (Sankyo Laboratory Service Corporation, Toyama, Japan). The procedure to produce the CD38^−/−^ mice was described previously (Kato et al. 1999). The offspring of wild‐type and CD38^−/−^ mice were born in our laboratory colony. Pups were weaned at 21–28 days of age and housed in same‐sex groups of five animals until pairing. A male and female of each genotype were paired and kept in a nursing cage in our laboratory under standard conditions (24°C; 12‐h light/dark cycle, lights on at 08:00) with food and water provided ad libitum. All the animal experiments were performed in accordance with the Fundamental Guidelines for the Proper Conduct of Animal Experiments and Related Activities in Academic Research Institutions under the jurisdiction of the Ministry of Education, Culture, Sports, Science, and Technology of Japan and were approved by the Committee on Animal Experimentation of Kanazawa University.

### Paternal retrieval test

Virgin males and females of identical genotypes were paired at 45–55 days. A single male and a single female were continuously housed together in a standard mouse maternity cage from the mating period to the delivery of pups and then to postnatal day 3–5. All the family units consisted of a new sire and dam and their first litter of each genotype, and all were experimentally naive. 30 min before starting the experiment, the cages with families were placed into the experimental room for habituation. After habituation, the sire received a single intraperitoneal injection of 0.3 mL of phosphate‐buffered saline (PBS) or 0.3 mL of OT (1 mL per 100 g of body weight), LOT‐1, LOT‐2, or LOT‐3 at a concentration of 100 ng/mL dissolved in PBS. Thirty minutes after injection, the sire and dam were placed for 10 min in a clean cage with new woodchip bedding, but the pups were left in the nest in the original cage. Five pups were randomly selected from the litter and placed individually at a site remote from the nest in the original cage. The sires were returned to the original home cage in the presence of their five biological pups to assess parental behavior. Parental retrieval behavior (latency to retrieve the first pup and time for retrieving all 5 pups) was examined for 10 min following reunion. The behavioral tests were carried out in a randomly mixed sequence of experimental groups. Experiments were usually performed between 10:00 and 15:00. The main experimental design in ICR and CD38^−/−^ mice was described previously (Akther et al. 2013; Liu et al. 2013). We also observed other parental behaviors (grooming, crouching, and huddling) as defined by Gubernick and Alberts (1987). Animals in this and subsequent experiments were tested only once.

### Cell culture transfection and construction of expression plasmids

Plasmid constructs were described previously (Amina et al. 2010). Human embryonic kidney HEK‐293 cells were maintained in Dulbecco's Modified Eagle's Medium (DMEM) supplemented with 10% fetal bovine serum (FBS) and Geneticin G418 100 *μ*g/mL at 37°C in a humidified atmosphere of 95% air and 5% CO_2_. Cells were grown in culture dishes to 80–90% confluence and transfected with the expression plasmid pcDNAHOXTR‐376R or pcDNA3(+) (Invitrogen, Carlsbad, CA) (MOCK‐transfected cells), using FuGENE HD Transfection Reagent (Roche, Basel, Switzerland) in accordance with the manufacturer's instructions.

### [Ca^2+^]_i_ measurement

We measured [Ca^2+^]_i_ using the fluorescent Ca^2+^ indicator fura‐2‐acetoxymethyl ester (fura‐2/AM). Transfected HEK‐293 cells were loaded with fura‐2/AM to a final concentration of 1 *μ*mol/L in complete medium and incubated at 37°C as reported previously (Ma et al. 2013). After 30‐min loading, the cells were washed three times with HEPES‐buffered saline (HBS) solution (145 mmol/L NaCl, 5 mmol/L KCl, 1 mmol/L MgCl_2_, 20 mmol/L HEPES‐NaOH, 2 mmol/L CaCl_2_, 20 mmol/L glucose, pH 7.4). The fluorescence of the cells loaded with fura‐2/AM was then measured at 37°C, at the determined sites, through a pinhole (10–20 *μ*m in diameter). We used alternating excitation wavelengths of 340 and 380 nm in a Ca^2+^ microspectrofluorometric system (IX‐73 Model; Olympus, Tokyo, Japan) and Metafluor software (Molecular Devices, Sunnyvale, CA), as described previously (Hashii et al. 2000). The Ca^2+^ emission was detected every 5 sec for 5 min after application of PBS, OT, or analogs. The ratio of fluorescence at 340 nm and 380 nm (F_340_/F_380_) was used to determine [Ca^2+^]_i_. All data were normalized to the baseline fluorescence (F_0_) recorded 10 s before addition, and given as F/F_0_ where F – maximal ratio of fluorescence after drug's application. OT and analogs for experiments were diluted in 50% ethanol to a concentration of 10^−3^ mol/L and then diluted in distilled water to obtain the required concentrations.

### Measurement of OT in plasma and CSF

To estimate the effects of our injections on circulating plasma OT levels, ICR male mice (*n* = 4–7) were injected intraperitoneally with vehicle, OT, or LOT‐1 (100 ng/kg in 0.3 mL of saline). Thirty minutes or 24 h later, mice were anesthetized by an intraperitoneal injection of pentobarbital. Blood samples of 0.1 mL were collected by cardiac puncture in EDTA and aprotinin‐coated capillaries and centrifuged at 1600*g* for 15 min at 4°C. Plasma samples were collected and stored at –80°C until use.

Mouse CSF was obtained according the techniques described by Jin et al. (2007). ICR male mice were 6–8 weeks old (*n* = 6–8) were injected intraperitoneally with vehicle, OT, or LOT‐1 (100 ng/kg in 0.3 mL of saline). Thirty minutes or 24 h later, the mice were anaesthetized with intraperitoneal injection of pentobarbital. The skin over the posterior neck was removed by two incisions, the first in the midline from the low cervical area to the anterior cranium and the second across the craniocervical junction. Subcutaneous tissue and nuchal muscles were exposed, sectioned along the rim of the occipital bone and then removed laterally unveiling the glistening clear arachnoid membrane overlying the cisterna magna. A micropipette was guided into the cisterna magna.

The micropipette was constructed by cutting a 30‐gauge needle with scissors to approximately 6–7 mm. The needle was then inserted 4–5 mm into the lumen of a 10 *μ*L micropipette. CSF samples were obtained between 8.00 and 11.00 am, and usually 5 *μ*L of clear CSF was obtained. The CSF samples were kept on ice and assayed immediately for OT.

Determination of OT was performed using a 96‐plate commercial OT‐ELISA kit (Enzo Life Sciences, Farmingdale, NY), as described previously (Jin et al. 2007; Lopatina et al. 2011). Protein content was determined using a Bio‐Rad protein assay kit and bovine serum albumin assay standard (Bio‐Rad, Hercules, CA).

### Statistical analysis

Two‐tailed Student's *t* tests were used for single comparisons between two groups. The rest of the data were analyzed by one‐way or two‐way analyses of variance (ANOVA) for two or three components, respectively. Post hoc comparisons were performed only when the main effect showed statistical significance. *P*‐values of the multiple comparisons were adjusted using Bonferroni's correction. All data from in vivo and in vitro studies are shown as means ± standard error of the mean. In all analyses, *P* < 0.05 was taken to indicate statistical significance. All the analyses were performed using the STATA data analysis and statistical software (Stata Corp. LP, College Station, TX). For the calculation and construction of EC_50_ graphs, GraphPad Prism 6 was used (GraphPad Software, La Jolla, CA).

## Results

### Structure of oxytocin analogs

**Figure 1:**
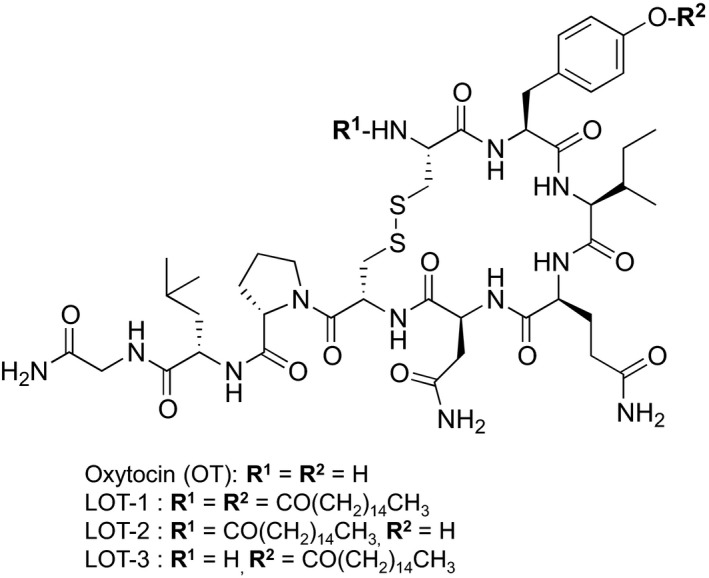
Chemical structures of OT and its lipidated analogs. LOT‐2 has conjugation of one palmitoyl group at the amino group of the Cys^1^ residue, LOT‐3 has conjugation of one palmitoyl group at the phenolic hydroxyl group of the Tyr^2^ residue, and LOT‐1 has conjugation of palmitoyl groups at both positions. Molecular weights: OT – 1007, LOT‐1 – 1483, LOT‐2 – 1245, LOT‐3 – 1245. OT, oxytocin.

The detailed structures of the three lipidated OT analogs are presented in Figure 1. The main difference between the native OT molecule and the LOTs is the presence of palmitoyl acid at the amino group of the Cys^1^ residue for LOT‐2, the phenolic hydroxyl group of the Tyr^2^ residue for LOT‐3 and both for LOT‐1.

### Agonist‐induced elevation in Ca^2+^ concentrations

**Figure 2:**
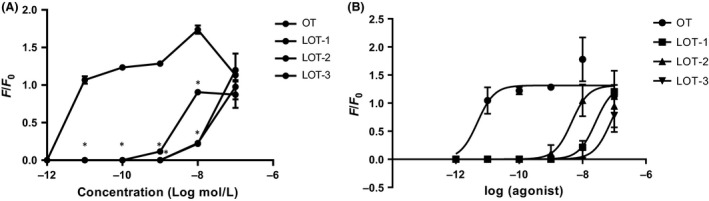
Microfluorometric recording from fura‐2‐loaded HEK cells expressing human oxytocin receptors. (A) Average peak value of [Ca^2+^]_i_ (F/F_0_ ratio) in response to various concentrations of OT and LOTs in HEK‐293 cells transfected with pcDNAHOXTR‐379R. Cells were incubated with Ca^2+^‐containing solution and chemicals were applied at the indicated equilibrium concentrations. Data are means ± SEM (*n* = 25–64 cells in three independent cultures). Statistical significance was evaluated by one‐way ANOVA with Bonferroni's correction, *P < 0.01, compared with OT. (B) EC_50_ shift. Each plot shows the percentage maximum response of [Ca^2+^]_i_ at the initial peak after treatment with OT or analogs. OT, Oxytocin.

We first examined the effect of the LOTs on the increase in [Ca^2+^]_i_ in HEK‐293 cells expressing the common type of human OT receptor (hOXTR). We applied 2 *μ*L of the test solutions at concentrations ranging from 10^−12^ to 10^−7^ mol/L at equilibrium in the culture medium. At a concentration of 10^−12^ mol/L OT, no initial rise was observed. Elevation from the pretreatment baseline level began at 10^−11^ mol/L OT (Fig. 2A), and the maximum initial transient increase was obtained at 10^−8^ mol/L OT (F/F_0_ = 1.737 ± 0.054; *n* = 25). OT at 10^−7^ mol/L resulted in significantly smaller [Ca^2+^]_i_ increases, with a reverse U‐shaped dose–response curve.

Little or no rise in [Ca^2+^]_i_ was observed with any of the three OT analogs at a concentration of 10^−9^ mol/L. At 10^−8^ mol/L, LOT‐2 showed significant and maximum elevation, whereas LOT‐3 and LOT‐1 showed relatively smaller responses: the ratio (F/F_0_) of [Ca^2+^]_i_ was 0.905 ± 0.014 (*n* = 25) for LOT‐2, 0.226 ± 0.031 (*n* = 5) for LOT‐1, and 0.218 ± 0.031 for LOT‐3 (*n* = 25). At 10^−7^ mol/L, OT and the three analogs exhibited elevation of [Ca^2+^]_i_ to almost the same level (Fig. 2A). Two‐way ANOVA indicated that the three LOTs induced significantly lower increases in the initial [Ca^2+^]_i_ peak than OT at concentrations from 10^−11^ to 10^−8^ mol/L (*n* = 25–63, *F*_*3,257*_ = 259.12, *P* < 0.05). All responses were significantly different from that of OT according to Bonferroni's post hoc tests (*P* < 0.05) (Fig. 2A).

Table 1 shows the percentage [Ca^2+^]_i_ of the lipidated analogs at 10^−8^ mol/L: OT was set to 100%, followed by LOT‐2, LOT‐3, and LOT‐1. The *EC*_*50*_ for OT was 10^−11.37 ^mol/L. The responses are replotted at a value of 10^−7^ mol/L in Figure 2B. It was difficult to calculate *EC*_*50*_ for OT analogs because there were fewer data points and the effect was not saturated. The *EC*_*50*_ shift, however, could be calculated. The *EC*_*50*_ ratio over OT (as 1) was 1342 ± 761 (*n* = 6) for LOT‐2, 5787 ± 3657 (*n* = 6) for LOT‐1, and 8828 ± 5474 (*n* = 6) for LOT‐3, respectively.

**Table 1:** Average elevation of intracellular free Ca^2+^ concentrations in HEK‐293 cells expressing *pcDNAHOXTR‐376R* stimulated with agonists

Tested drugs	Percentage of maximum Ca^2+^ mobilization
	Mean ± SEM, *n* = 25
OT	100
LOT‐1	12.6 ± 1.8 a ^**,**^ c
LOT‐2	45.5 ± 0.83 a ^**,**^ b
LOT‐3	13.1 ± 1.8 a ^**,**^ c

Values represent percentage of drug responses at 10^−8^mol/L, relative to that of OT as 100%. OT, Oxytocin; HEK, human embryonic kidney.

One‐way ANOVA with Bonferroni's correction:

a *P* < 0.05 compared with OT.

b *P* < 0.05 compared with LOT‐1.

c *P* < 0.05 compared with LOT‐2.

No [Ca^2+^]_i_ response associated with OT or the analogs at concentrations of 10^−8^ mol/L and 10^−9^ mol/L was observed in experiments with MOCK‐cells. These data indicated that the three lipidated analogs were >1000‐ to 10,000‐fold less active than native OT in OT receptor activation that triggers Ca^2+^ mobilization from intracellular Ca^2+^ pools. The results also suggest that OT receptor stimulation differs slightly depending on the site of palmitoylation: LOT‐2, with palmitoyl acid at the cysteine residue, was slightly more effective than those with palmitoyl acid at the tyrosine residue (LOT‐1 and ‐3).

### Paternal retrieval test

**Figure 3:**
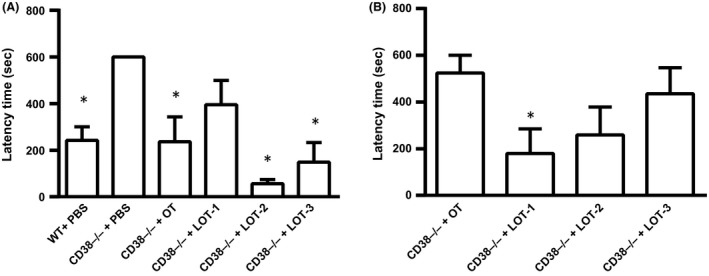
Pup retrieval by sires. Average latency time in seconds to start retrieval of biological pups by wild‐type (WT) or CD38^−/−^ sires at 30 min (A) and 24 h (B) after single subcutaneous injection of PBS, OT, or LOTs (30 ng/mouse), *n* = 5–6 for each test. One‐way ANOVA followed by Bonferroni post hoc test was performed for 30 min (*F*_5,39_ = 7.60, *P* = 0.0001) and for 24 h (*F*_5,40_ = 5.35 *P* = 0.0007). *P < 0.05, Bonferroni's post hoc test compared with CD38^−/−^ treated by PBS. OT, Oxytocin; PBS, phosphate‐buffered saline.

As reported previously (Akther et al. 2013; Liu et al. 2013), 60–80% of wild‐type sires with or without intraperitoneal injection of PBS displayed parental retrieval behavior (*n* = 16) with a mean latency of 242 ± 57 sec after 40 min of separation together with their mate dams in a novel cage for 10 min (Fig. 3A). In addition, approximately 60% demonstrated full paternal retrieval behavior. In contrast, CD38^−/−^ sires with PBS treatment (*n* = 9) failed to retrieve their pups, and thus had a latency of 600 sec. These phenotypes in wild‐type and CD38^−/−^ sires were relatively stable and constant.

CD38^−/−^ sires, 30 min after a single intraperitoneal injection of OT (100 ng/mL, 1 mL per 100 g of body weight), displayed retrieval behavior with an average latency of 236 ± 106 sec (*n* = 5), which was nearly identical to that of the wild‐type (Fig. 3A).

The pup retrieval latencies in CD38^−/−^ sires 30 min after a single intraperitoneal injection of 100 ng/mg (1 mL per 100 g of body weight) of LOT‐1, LOT‐2, or LOT‐3 were 395 ± 103 sec (*n* = 5), 56 ± 18 sec (*n* = 5), and 149 ± 84 sec (*n* = 5), respectively (Fig. 3A). One‐way ANOVA revealed significant differences: (*F*_5,39_ = 7.60, *P *<* *0.05). Bonferroni's post hoc tests in CD38^−/−^ mice indicated significant differences between the saline‐treated group and those treated with OT (*P *=* *0.001), LOT‐2 (*P* = 0.002), and LOT‐3 (*P* = 0.004), but no difference was observed for LOT‐1 (*P* = 0.282).

Next, we examined the long‐acting effects of LOTs, because LOT‐1 has long positive effects on social avoidance in CD157 knockout mice (Mizuno et al. 2015). As shown in Figure 3B, 24 h after injection in CD38^−/−^ sires, the average latencies of LOT‐1, LOT‐2, and LOT‐3 were 179 ± 106 s (*n* = 5), 259 ± 120 s (*n* = 5), and 436 ± 111 s (*n* = 5), respectively. In marked contrast, that of OT was 525 ± 75 s (*n* = 6). One‐way ANOVA revealed significant differences: (*F*_5,40_ = 5.35, *P* < 0.05); Bonferroni's post hoc test revealed a significant difference between saline and LOT‐1 (*P* < 0.05), whereas no significant differences were found for the other treatment groups.

**Figure 4:**
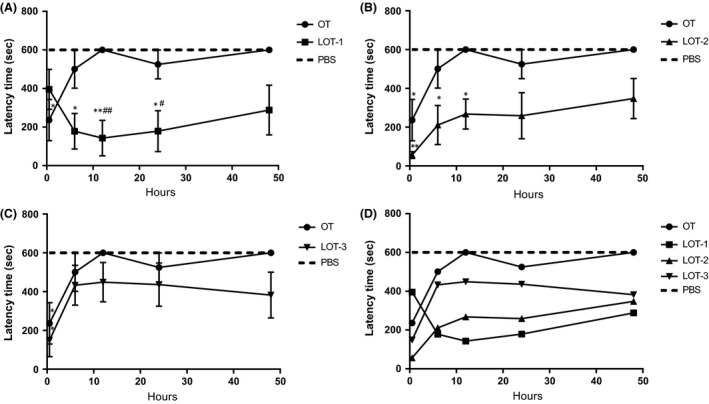
Time course in latency of pup retrieval behavior. Latency time in seconds to start pup retrieval in CD38^−/−^ sires treated with PBS (cross‐hatched lines) and OT (circles) or LOT‐1 (squares, A), LOT‐2 (triangles, B), and LOT‐3 (inverted triangles, C). Data show average latencies at various time points after injection, as in Fig. 3. (D) Combined displays of time course in latency in sires’ pup retrieval behavior after injection of OT or LOTs without error bars. One‐way ANOVA followed by Bonferroni's post hoc test performed for 6 h (*F*_5,40_ = 5.60, *P* = 0.0005), for 12 h (*F*_5,43_ = 7.75 *P* = 0.0001), and for 48 h (*F*_5,39_ = 4.97, *P* = 0.0013). *P < 0.05, **P < 0.01 compared with PBS; ^#^ *P* < 0.05, ^##^ *P* < 0.01 compared with OT treatment. *N* = 5–6 mice for each data point. OT, Oxytocin; PBS, phosphate‐buffered saline.

We examined the time course over 48 h, which was not performed in the previous study (Mizuno et al. 2015). LOT‐1 showed significant effects on latency at 6, 12, and 24 h after injection into CD38^−/−^ sires, whereas at 48 h, the latency was shorter than that with OT or PBS, but the difference did not reach significance (Fig. 4A). LOT‐2 showed significant effects on latency at 30 min, 6, and 12 h, but after 24–48 h its activity was not significantly different from OT and PBS (Fig. 4B). LOT‐3 showed a significant effect on latency only at 30 min, with no significance between 6 and 48 h (Fig. 4C). For better comparison, the time courses of the four compounds over 48 h were replotted without error bars in Figure 4D. As seen in the figure, the time course of LOT‐3 resembles that of OT, whereas those of LOT‐1 and LOT‐2 are similar to each other. Two‐way ANOVA indicated significant effects of model (*F*_29,230_ = 5.49, *P* < 0.05), mouse strain (*F*_1,230_ = 91.39, *P* < 0.05), and treatment conditions (*F*_4,230_ = 22.08, *P* < 0.05). Bonferroni's post hoc tests showed that OT and LOT‐3 did not have effects compared with saline at 6 h (OT, 501 ± 99 s, *n* = 5 *P *>* *0.99; LOT‐3, 433 ± 102 sec, *n* = 5, *P *=* *0.53) and 12 h (OT, 600 ± 0 sec, *n* = 5, *P *>* *0.99 vs. LOT‐3, 449 ± 101 sec, *n* = 5, *P* = 0.281). LOT‐2 showed a significant effect at 6 h (211 ± 100 sec, *P* < 0.05, *n* = 5). The effect of LOT‐1 was significantly different from that of saline at 6 h (*P* < 0.05, *n* = 8) and 12 h (*P* < 0.05, *n* = 6).

The time required for retrieval of all five pups to the nest was measured as an additional parameter in parental retrieval behavior. Sires at 30 min after injection of OT and LOTs displayed retrieval behavior. Significant retrieval was observed only with OT (*P* < 0.05) and LOT‐2 (*P* < 0.01) according to one‐way ANOVA (*F*_5,33_ = 6.68, *P* < 0.05; Table 2). However, significant effects on time to retrieve all pups to the nest at 6, 12, and 24 h posttreatment were observed only for LOT‐1 compared with saline (*F*_5,36_ = 8.01, *P* < 0.05; *F*_5,38_ = 11.64, *P* < 0.05; *F*_4,34_ = 9.46, *P* < 0.05, respectively, by one‐way ANOVA). The results indicated that all the compounds lost activity 48 h after injection, and that the order of activity after 24 h was LOT‐1 > LOT‐2 > LOT‐3 > OT. These results suggested that LOT‐1 has the longest lasting effect between OT and the three OT analogs.

**Table 2:** Time to retrieve five pups at different time points after single injection of OT or LOT analogs in CD38^−/−^ sires

Tested drugs	Time (min) required to retrieve 5 pups to the nest (mean ± SEM)				
	30 min	6 h	12 h	24 h	48 h
OT	302 ± 100^a^	523 ± 77	600 ± 0	575 ± 25	600 ± 0
LOT‐1	494 ± 106	229 ± 82^b^	242 ± 80^b^	242 ± 91^b^	436 ± 101
LOT‐2	157 ± 34^b^	419 ± 75	496 ± 59	379 ± 100	444 ± 96
LOT‐3	339 ± 117	530 ± 70	479 ± 82	457 ± 101	501 ± 99
PBS	600 ± 0	600 ± 0	600 ± 0	600 ± 0	600 ± 0

The times required to retrieve five pups into the nest during 10 min at five different time points after injection are listed (*n* = 144). One‐way ANOVA with Bonferroni's correction between CD38^−/−^ mice treated with PBS (600 ± 0) and each row. OT, Oxytocin; PBS, phosphate buffered saline.

a *P* < 0.05 from PBS control.

b *P* < 0.01 from PBS control.

With regard to other parameters of paternal retrieval behavior, such as parental behavior score (Table S1), we obtained essentially similar results, in that LOT‐1 showed the longest‐acting effects. We did not find any differences in grooming, crouching, or huddling between test groups.

### Measurement of oxytocin in plasma and CSF

**Figure 5:**
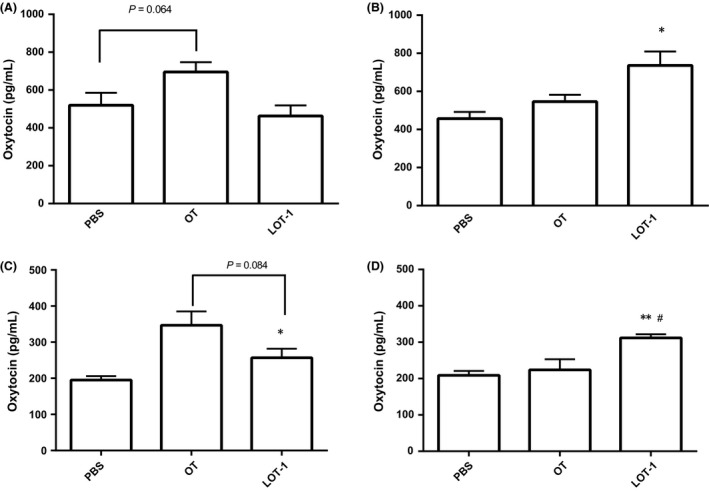
Plasma and cerebro‐spinal fluid oxytocin levels after injection of chemicals. Concentrations of OT in the plasma after 30 min (A) and 24 h (B), in cerebro‐spinal fluid after 30 min (C) and 24 h (D) in WT mice intraperitoneal treated with PBS, OT, or LOT‐1. One‐way ANOVA followed by Bonferroni's post hoc test revealed *F*_2,25_ = 4.31, *P* = 0.0247 for A (*n* = 5–8), *F*_2,12_ = 5.10, *P* = 0.025 for B (*n* = 4–7), *F*_2,17_ = 7.28, *P *=* *0.0052 (n = 6–8) for C, *F*_2,15 _= 8.57, *P *=* *0.0033 (n = 6). *P < 0.05, **P <* *0.01 compared with PBS‐treated group, #*P *<* *0.05 compared with OT‐treated group. OT, Oxytocin; PBS, phosphate‐buffered saline.

To explain the long‐acting effects of LOT‐1, we determined the levels of OT in the plasma and CSF of WT mice after three types of treatment, that is, PBS, OT, or LOT‐1, at two time points: 30 min and 24 h after injection. As shown in Figure 5A, 30 min after injection, the plasma level of OT was slightly higher in the OT‐treated group compared with the PBS‐treated group (PBS, 519 ± 65 pg/mL vs. OT, 695 ± 52 pg/mL) according to one‐way ANOVA with Bonferroni's test (*n* = 5–8, *F*_2,25_ = 4.31, *P* = 0.064). LOT‐1‐treated mice did not show any difference (463 ± 57 pg/mL, *P* > 0.99). In contrast, measurement after 24 h showed the opposite result (Fig. 5B). One‐way ANOVA showed a significant effect of treatment conditions (*n* = 4–7, *F*_2,12_ = 5.10, *P* < 0.05). Post hoc comparisons showed that LOT‐1 had a significantly higher effect on plasma OT level than PBS (PBS, 457 ± 35 vs. LOT‐1, 736 ± 73 pg/mL, *P* < 0.05), whereas OT showed no difference at this time point (PBS, 457 ± 35 vs. OT, 547 ± 39, *P* = 0.480).

As shown in Figure 5C, 30 min after injection, the level of OT in the CSF was significantly higher in the OT‐treated group compared with the PBS‐treated group (PBS, 195 ± 11 pg/mL vs. OT, 347 ± 38 pg/mL, Bonferroni comparison *P *<* *0.05), whereasLOT‐1‐treated mice did not show any difference (257 ± 25 pg/mL, *P *=* *0.349). One‐way ANOVA (*n* = 6–8, *F*_2,17_ = 7.28, *P *=* *0.0052) demonstrated a significant effect on the treatment conditions. Measurement after 24 h showed the opposite result (Fig. 5D); one‐way ANOVA showed a significant effect on the treatment conditions (*n* = 6, *F*_2,15_ = 8.57, *P *=* *0.0033). Post hoc comparisons showed that LOT‐1 had a significantly higher effect on plasma OT level than PBS (PBS, 209 ± 12 vs. LOT‐1, 312 ± 9.9 pg/mL, *P *<* *0.01), whereas OT showed no difference at 24 h posttreatment (PBS, 209 ± 12 vs. OT, 224 ± 29, *P *>* *0.99) LOT‐treated group also demonstrated significant difference with the OT‐treated group (*P *<* *0.05).

The data suggest that OT injection has an acute effect on the elevation of plasma and CSF OT levels, whereas LOT‐1 did not elevate the OT level in the short‐term but had a long‐lasting effect.

## Discussion

We characterized the pharmacological properties of palmitoyl OT. OT and three OT analogs with one or two palmitoyl moieties showed agonist‐induced [Ca^2+^]_i_ elevation in HEK‐293 cells expressing hOXTR‐376R. OT receptors showed activity at concentrations from 10^−11^ mol/L to 10^−7^ mol/L with a peak at 10^−8^ mol/L. Although the effective concentration ranges were slightly wider than those in our previous report (Ma et al. 2013), this discrepancy can be explained by differences between the methods. In our previous study (Ma et al. 2013), we used transiently transfected HEK‐293 cells, whereas stably transformed cells were used in this study. The elevation in [Ca^2+^]_i_ elicited by LOT‐2 at 10^−8^ mol/L was half that of OT, LOT‐1 and LOT‐3 elicited only slight increases in [Ca^2+^]_i_. The *EC*_*50*_ shift showed that LOT‐1 and LOT‐3 had more than 5000‐fold lower activity than OT, and the activity of LOT‐2 was nearly 1000‐fold less than that of OT. These functional results suggest that the addition of palmitoyl groups to the parental OT molecule likely blocked the ability to activate OT receptors. The structural changes on LOT likely decreased binding capacity for the receptor. Interestingly, the small difference in activity between LOT‐2 and LOT‐1/LOT‐3 implicates the modification location. Although LOT‐1 has twos palmitoyl groups and LOT‐3 has one, both compounds have a palmitoyl chain at the phenolic hydroxyl group of the Tyr^2^ residue, whereas LOT‐2 has a palmitoyl chain only at the Cys^1^ residue. Although the palmitoylation results in an ester at Tyr^2^ and an amide at Cys^1^ this factor probably did not affect the immediate in vitro reactions. However, the different chemical motifs may affect the stability in the body; these relationships are summarized in Table 3. An important limitation of this study is that the affinities of agonists to the OT receptors were not determined directly by receptor binding experiments. These experiments might help to determine whether the LOT to the receptors that are sterically blocked from binding. Such experiments, using concurrent isotope binding, are currently underway in our laboratory.

**Table 3:** Summary of molecular structures and activities of oxytocin and its analogs

	Number of added palmitoyl groups	Position	[Ca^2+^]_i_	Retrieval behavior	
				30 min	24 h
OT	0		++++	+	0
LOT‐1	2	cys^1^, tyr^2^	1/8000≈0	0	+++
LOT‐2	1	cys^1^	1/1000 +	++	0
LOT‐3	1	tyr^2^	1/5000≈0	+	0

The table shows summarized data regarding structural differences of LOT analogs, in vitro [Ca^2+^]_i_ measurements, and results of parental behavior experiments in CD38^−/−^ mice. OT, Oxytocin; LOT, lipo‐oxytocin.

It is possible that the lower activity of the LOTs may have been due to contamination by OT during purification. However, this contamination is unlikely because the compounds were purified by silica gel column chromatography. If such effects were due to contamination, the resulting effects would have been equal between LOTs.

We successfully estimated the in vivo effects by testing parent–pup interaction, which is one of the most prominent social interactions (Liu et al. 2013) and is lost in CD38^−/−^ mice (Akther et al. 2013). Although there were slight differences in effects with different compounds, parameters, and time points, our compounds can be split into two classes based on the in vivo results. The first class includes OT, LOT‐2, and LOT‐3, which have positive effects at 30 min but none at 24 h. The second consists of LOT‐1, which showed the opposite effect. According to these findings, LOT‐1 could have a long‐lasting effect superior to that of native OT or other lipidated analogs. We estimate that LOT‐1 would have a longer half‐life in mice, and that breakdown would stem from cleavage of LOTs into OT. Unfortunately, we were unable to identify LOTs in body fluids, because the OT ELISA kit was unable to detect the synthetic LOTs. For this same reason, we were unable to report the rate and efficiency at which LOTs penetrate the BBB. Even with mass spectrometry analysis, we failed to detect LOTs in plasma extracts in our preliminary experiments.

In the current experiments on LOT constructs, we applied the use of “prodrug” strategies by attaching fatty acid chains to decrease systemic clearance, thereby obtaining longer pharmacodynamics. However, this does not imply that our strategy is superior than other possible strategies, such as (1) rapid systemic clearance of natural peptides by introducing D‐amino acids or cyclization into the plasma compartment, (2) use of drug depot strategies to create slow release, and (3) the use of specific targeting constructs such as linkers or antibodies. Further work is necessary to modulate the pharmacodynamics of OT and the LOTs.

We obtained identical in vivo data for LOT‐1 in both CD157 (Mizuno et al. 2015) and CD38 knockout mice. CD157^−/−^ mice showing severe anxiety‐related and depression‐like behaviors are considered to be a model of the psychiatric nonmotor symptoms of Parkinson's disease and of the fear and social avoidance seen in ASD (Lopatina et al. 2014). A recent study indicated an association between the CD157/BST gene and risk of ASD in the Japanese population (Yokoyama et al. 2015). Therefore, CD157 knockout mice may be a model of ASD, just as CD38 knockout mice are a model of ASD with defects in social interaction (Higashida et al. 2012). LOT‐1 was shown to exhibit long‐lasting recovery effects in social avoidance in CD157^−/−^ mice and in parental behavior in CD38^−/−^ mice. Thus, LOT‐1 is of vital importance as a potent and durable OT analog that is likely to have significant advantages over OT itself in therapy for psychiatric disorders, such as ASD.

Table 3 shows that the results of the in vitro Ca^2+^ reaction do not completely parallel those obtained in vivo. Changes in [Ca^2+^]_i_ levels occurred in the order of milliseconds to seconds, whereas the behavioral observations occurred over minutes to hours. These findings suggest that behavior may be elicited by continuous activation of OT receptors, which can explain why LOT‐1 has long‐acting effects without immediate in vitro Ca^2+^ responses. Moreover, it is necessary to consider the metabolism of analogs in blood and penetration of the BBB. Alternatively, OT cleaved from LOT in the brain continuously stimulates OT‐induced OT release (Zhong et al. 2016). Further detailed analyses are necessary.

With regard to the long‐lasting effect of LOT‐1, we measured CSF and plasma OT levels at two time points after drug administration (30 min and 24 h). At 30 min after OT injection, the level of OT in plasma and CSF was slightly higher than that in PBS‐treated controls, whereas no such difference was observed following injection of LOT‐1. Interestingly, after 24 h, mice treated with LOT‐1 showed elevated plasma and CSF OT levels, whereas OT‐treated animals did not. The data support the suggestion that LOT‐1 is metabolized in the blood and transformed to OT. If long‐term conversion to OT is the main mechanism underlying the behavioral effects of LOT‐1, this could explain the long‐lasting effect of LOT‐1 as well as its slight or absent acute effect.

The effects of peripherally applied OT on socioemotional behavior, including social memory and parental behavior, may be partly due to peripheral effects on the sympathetic nervous system (Porges 2001; Gutkowska and Jankowski 2012). However, we believe that LOT‐1 is incorporated into the brain, as it has been shown that the cerebrospinal OT concentration is increased after subcutaneous injection of OT into ICR mice (Jin et al. 2007; Hofmann et al. 2015), with no direct evidence of efficient OT uptake into the brain. It is important to determine whether this novel agonist is indeed taken up into the brain via the BBB.

In conclusion, we applied a lipidation strategy (Kang and Park 2000) using palmitoyl acid to improve the drug‐like properties of OT. Lipidation of OT at two positions successfully yielded LOT‐1 a peptide drug with long‐lasting activity, as suggested by Varamini and Toth (2013). Although LOT‐2 and LOT‐3 exhibited less beneficial activity compared to LOT‐1, contrary to our initial expectations, they yielded insight into the mechanisms underlying the effects of LOT‐1. Based on our results, LOT‐1 is potentially useful for psychiatric diseases, including ASD. Before it can be applied in phase III clinical trials, however, it will be important to determine the pharmacodynamics in the body, procedures for mass production, material stability, toxicity, and pharmacokinetic data.

## Author Contributions

Stanislav M. Cherepanov, Shigeru Yokoyama, Satoshi Shuto, and Haruhiro Higashida participated in research design. Stanislav M. Cherepanov, Shigeru Yokoyama, Akira Mizuno, Wataru Ichinose, Olga Lopatina, Anna A. Shabalova, Alla B Salmina, Yasuhiko Yamamoto, and Hiroshi Okamoto conducted experiments. Stanislav M. Cherepanov, Akira Mizuno, Wataru Ichinose, and Satoshi Shuto contributed new reagents or analytic tools. Stanislav M. Cherepanov, Olga Lopatina, Anna A. Shabalova, and Haruhiro Higashida performed data analysis. Stanislav M. Cherepanov, Shigeru Yokoyama, Satoshi Shuto, and Haruhiro Higashida wrote or contributed to the writing of the manuscript:

## Disclosure

None declared.

## Supporting information


**Table S1.** Parental behavior in CD38^−/−^ sires after single injection of OT or LOT‐analogs.
