# Supplementary material for: Structure‐specific effects of lipidated oxytocin analogs on intracellular calcium levels, parental behavior, and oxytocin concentrations in the plasma and cerebrospinal fluid in mice
Source: Pharmacol Res Perspect. 2017 Jan 17;5(1):e00290. doi: 10.1002/prp2.290 (PMC5461640; doi:10.1002/prp2.290)
Supplement: Supplementary file 1 — Table S1. Parental behavior in CD38−/− sires after single injection of OT or LOT‐analogs. [file PRP2-5-e00290-s001.pdf]

Supplementary Table 1

Parental behavior in CD38-/- sires after single injection of OT or LOT-analogs. Parental scores.

Tested Drugs	Parental scores mean±sem				
	**30 min**	**6 hours**	**12 hours**	**24 hours**	**48 hours**
OT	**6±1.98****	2±2.0	0±0	1.66±1.66	0±0
LOT-1	3.8±2.01	**6.62±1.56****	**6.5±1.61****	**6.8±1.74****	3.4±1.88
LOT-2	**9.2±0.37****	**5.8±1.95***	4.5±1.48	**6.4±2.06***	3.4±1.72
LOT-3	**9.2±0.58****	3.2±2.05	2.83±1.79	3.2±1.98	2.8±1.85
PBS	0±0	0±0	0±0	0±0	0±0

One-way ANOVA test with Bonferroni correction between CD38-/- mice treated by PBS (0±0) and each of the rows was used for comparison.* indicates p value <0.05, ** indicates p value <0.01.
